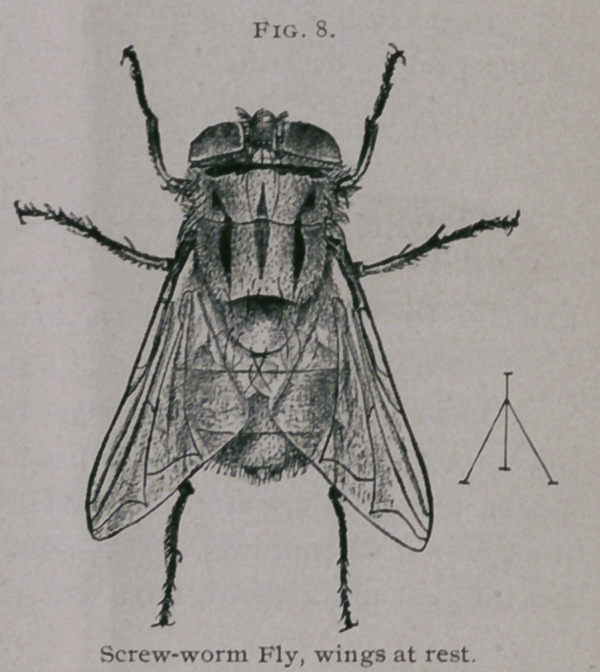# The Screw-Worm

**Published:** 1891-01

**Authors:** M. Francis


### THE SCREW-WORM.

BY M. FRANCIS, D.V.M.

SEVERAL years ago I published some notes on the screw-worm,[2] giving only such facts as I had witnessed, and remedies which I had employed. After two years of study and observation, during the summer months of which I have seen cases of the parasite almost daily, it is thought advisable to give a more exhaustiv- report of the life history of the insect, together with illustrations (see note 1) and description (see note 2) for identification.

The screw-worm is the larva or maggot of a dipterous insect (*Lucilia macellaria*) that is very common in this portion of the country during the summer, and is parasitic on man and animals.

The mature insect ("Imago") is a fly, a trifle larger than our

[2] First Annual Report of the Texas Agricultural Experiment Station.

ordinary house fly, with a yellow head and three dark longitudinal lines on the thorax. The abdomen is yellowish-green. The fly lays its eggs in wound sores, and even in the natural openings of man and animals.

In "Animal Parasites and Messmates," by Von Benenden, page 119, there appears the following: "There is another fly in Mexico which is dangerous to man; it is known by the name of *Musca hominivora*, or more correctly, *Lucilia hominivora*. Vercammer, a military surgeon of the Belgian army, relates that a soldier in Mexico had his glottis destroyed, and the sides of the roof of his mouth rendered ragged and torn, as if a cutting-punch had been driven into those organs. This soldier threw up with his spittle more than two hundred larvæ of this fly."

Prof. James Law, of Cornell University, in a letter to the writer, says: "Under the name of *Lucilia hominivora* it is said to be very destructive to the French convicts at Cayenne, the fly depositing its eggs in the mouth and nostrils during sleep."

No cases in man have fallen under the personal observation of the writer.

The fly seems to be well distributed over the American Continent, for Dr. Williston, of Yale College, writes that "it occurs everywhere from Canada to Patagonia." Although so generally distributed, only in Texas does it bear an economic importance in the United States. Of all our domesticated animals cattle suffer the most from its ravages. They occur in wounds from horns, castrating, spaying, branding, dehorning, barbed wire injuries, and often where ticks have burst on the brisket, flank, or just behind the udder of cows. They often occur in the vulvæ of fresh cows, especially if there has been a retention of the placenta or afterbirth. Young calves are almost invariably affected in the navel and often in the mouth, causing the teeth to fall out. One case occurred in the first stomach (paunch, or rumen) that is worthy of mention: Last September the writer had occasion to kill a Jersey bull calf, probably two months old, that had screw-worms in both hind legs just above the hock joint. On opening the abdomen I found hair-balls in the stomach (rumen), and, to my surprise, about twenty-five fully matured screw-worms almost buried in the wall of that organ. I placed some of the worms in moist earth, and in ten or twelve days they hatched out genuine screw-worm flies. How did they come there? My opinion is that the calf licked the sores on his legs, and in doing so took in some eggs that hatched and developed in the stomach.

Horses and mules are not so often attacked. In them they are usually found in barbed wire injuries, and occasionally in the sheaths of horses, the vaginæ of mares, and the navels of colts.

Hogs are more liable to become affected than horses. They are frequently wounded by dogs and by fighting, or there may be barbed wire injuries, wounds from castration, etc.

Sheep are comparatively free from attacks unless injured by dogs.

In all animals alike, the eggs, after being laid by the fly, hatch into larvæ or so-called "worms." The exact length of time this requires seems to vary with circumstances. My present opinion is that, if the eggs are laid in a moist place and on a warm day, it requires less than one hour; whereas, if laid in a dry place they seem to dry up and lose their vitality.

FIG. 1.

Single egg, greatly enlarged.

FIG. 2.

Bunch of Eggs.

FIG. 3.

Larva ("Screw-worm").

The young larvæ when first hatched are small and easily overlooked. If they are hatched on the surface in a drop of blood from a ruptured tick, for instance, they attempt to perforate the skin, and if hatched in wounds they at once become buried out of sight. They seem to attach themselves by their heads, and burrow their way under the skin, completely devouring the soft flesh. Occasionally a few are seen moving from one place to another, but usually they remain fixed at one point. The worms grow steadily in size, and the hole in the flesh becomes larger every day. Sometimes the worms make tunnels, but not to any depth; they usually stay on the surface. They evidently produce considerable irritation, for the part is always swollen and constantly bleeding. This swollen, gaping appearance of the

FIG. 4.

Pupa, or Chrysalis.

FIG. 5.

Pupa case, showing broken end where fly emerged.

wounds, together with the constant discharge of blood, are characteristic of the presence of worms. It seems to require about a week for the worms to become fully grown. At that time they are about five-eighths to six-eighths of an inch long. They then leave the sore and go into the ground, where they pass their pupa state and hatch out as flies in from nine to twelve days. Of several hundred hatched out by the writer, the shortest time was nine days and the longest fourteen days, but in the majority of cases it required from nine to twelve days.

FIG. 6.

Side view of head and mouth parts.

FIG. 7.

Screw-worm Fly, wings expanded.

While the larvæ are thus developing the flies are constantly laying fresh eggs in the wounds, so that the young worms take the places of the matured ones, and thus keep up a constant and progressive loss of tissue. If the worms are not killed they eat constantly deeper, and often kill the animal. Sometimes the abdomen is opened and the bowels escape—as is especially liable in case of heifers spayed through the abdomen. At other times a tail is eaten off, or extensive caverns are made into the muscles.

FIG. 8.

Screw-worm Fly, wings at rest.

The treatment usually employed in these cases consists simply of killing the larvæ with cresylic ointment, calomel, chloroform, or carbolic acid. The selection of the most suitable remedy will vary somewhat with the location, character and extent of the sores. In some cases bandages are useful. In others the sores can be filled with oakum and a few stitches taken. All treatment should be supplemented by daubing the margins of the wound with pine tar to ward off the fly. A vast number of cases can be prevented by keeping cattle free from common cattle ticks.

### NOTES.

NOTE 1.—The illustrations used in this Bulletin have been prepared from the living insect in its different stages by Miss Freda Detmers, of the Ohio Experiment Station, at Columbus, Ohio.

NOTE 2.—Description by Clarence M. Weed, Entomologist to the Ohio Experiment Station, at Columbus, Ohio.

### COMPSOMYIA (LUCILIA) MACELLARIA.

IMAGO.—Length 10 mm. ($\frac{2}{5}$ inch); wing expanse 21 mm. ($\frac{4}{5}$ inch); color metallic bluish-green with golden reflections; thorax with three black longitudinal stripes; head, except central portion of eyes, yellow; legs black; wing veins black; wings transparent except near base, where they are slightly clouded. Entire body furnished with long black spinose hairs. Proboscis of medium length, with dilated tip.

LARVA.—Length 16 mm. ($\frac{3}{5}$ inch); diameter 4 mm. ($\frac{1}{5}$ inch). A whitish footless grub of shape represented in figure 3, with rows of stiff black bristles at each articulation.

PUPARIUM.—Length 10 mm. ($\frac{2}{5}$ inch); diameter 3 mm. ($\frac{1}{8}$ inch). Brown in color and of shape represented in figure 4.

EGG.—Length 1 mm. ($\frac{1}{25}$ inch). Cylindrical, with a longitudinal ridge on upper side. Whitish.

## Figures and Tables

**Fig. 1. f1:**
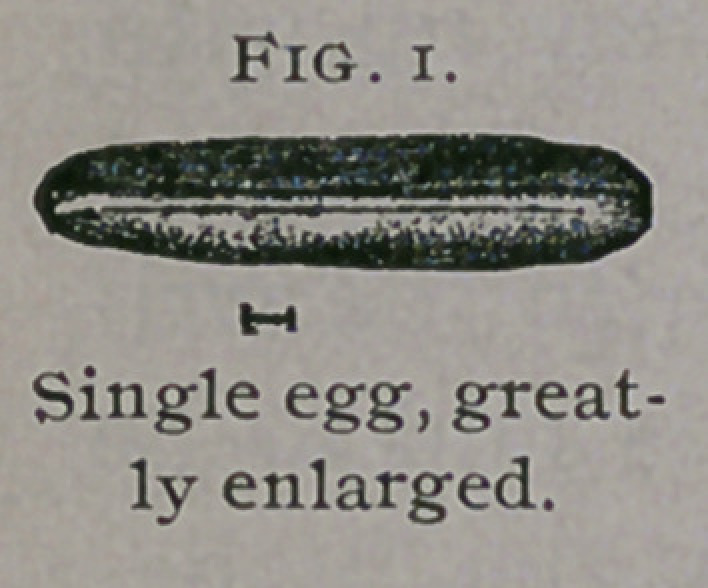


**Fig. 2. f2:**
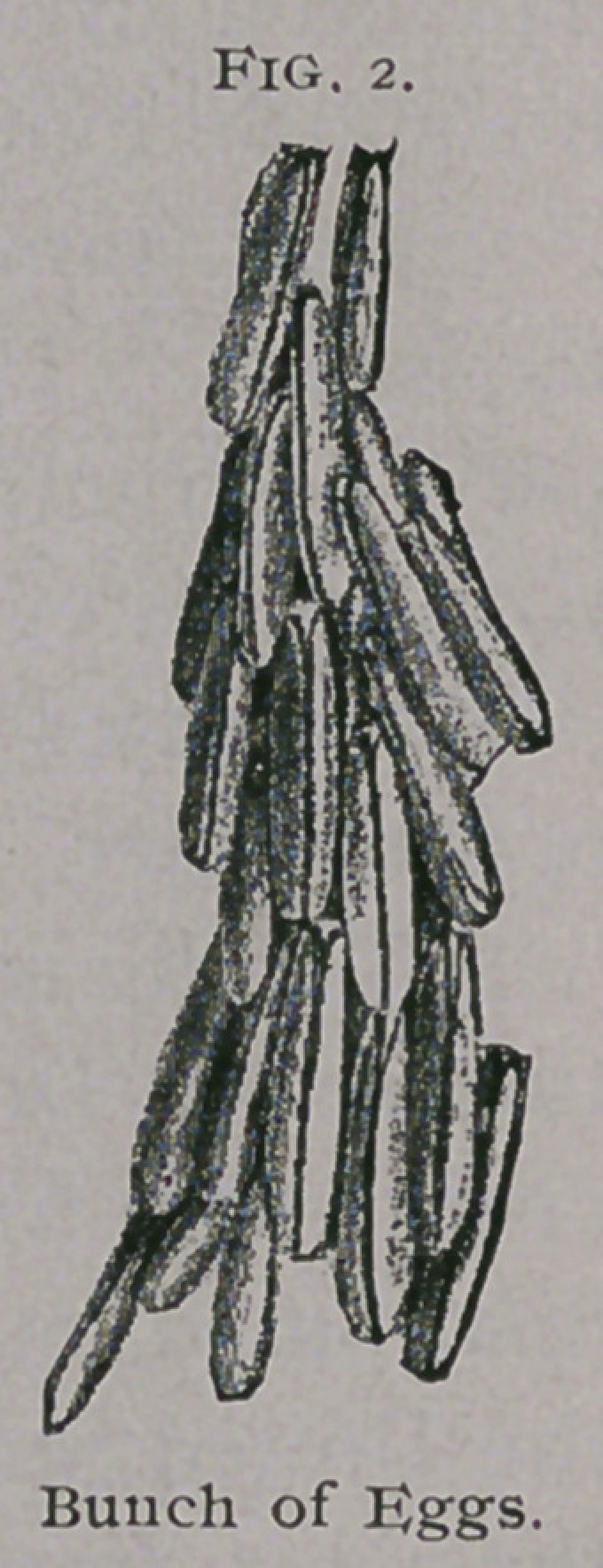


**Fig. 3. f3:**
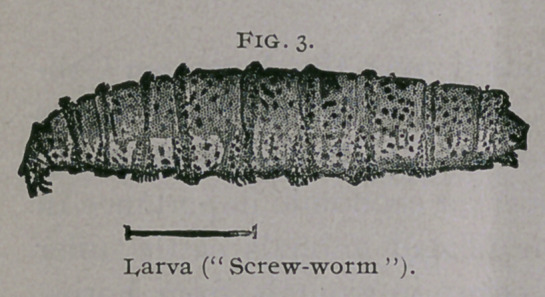


**Fig. 4. f4:**
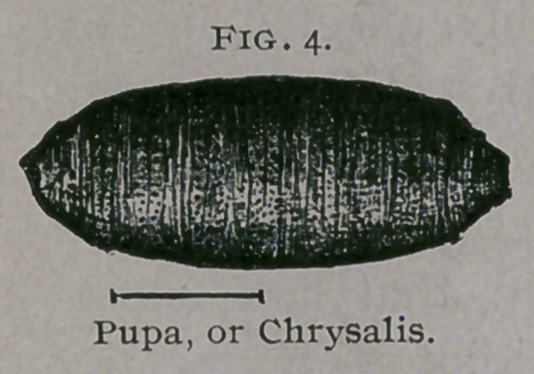


**Fig. 5. f5:**
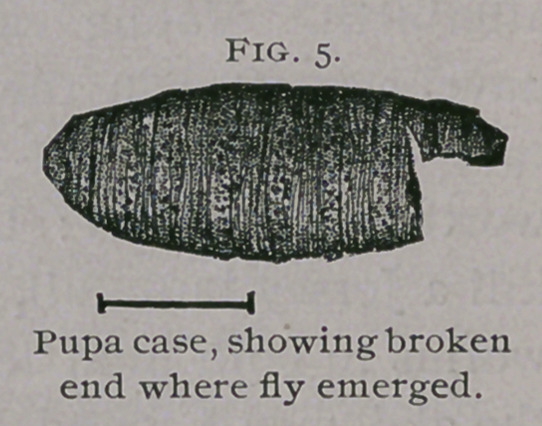


**Fig. 6. f6:**
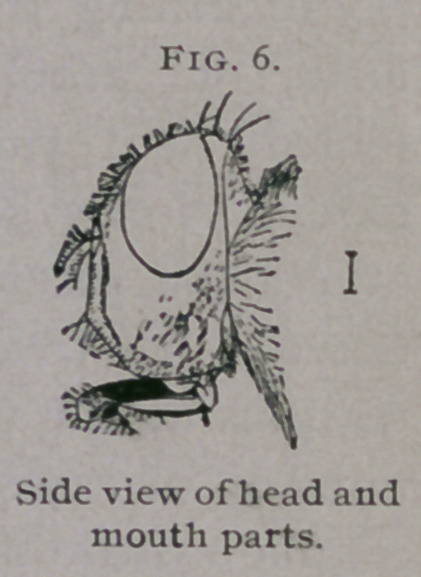


**Fig. 7. f7:**
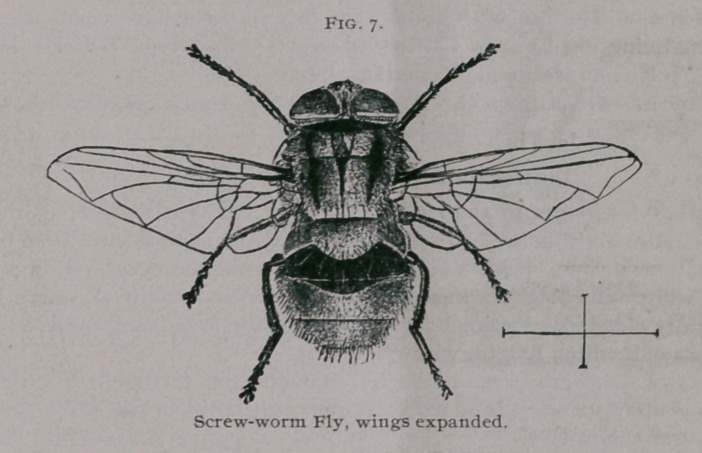


**Fig. 8. f8:**